# Non-canonical Eclosion Hormone-Expressing Cells Regulate *Drosophila* Ecdysis

**DOI:** 10.1016/j.isci.2020.101108

**Published:** 2020-04-27

**Authors:** Robert L. Scott, Fengqiu Diao, Valeria Silva, Sanghoon Park, Haojiang Luan, John Ewer, Benjamin H. White

**Affiliations:** 1 Laboratory of Molecular Biology, National Institute of Mental Health, NIH, Bethesda, MD 20892, USA; 2 Centro Interdisciplinario de Neurociencia, Universidad de Valparaiso, Playa Ancha, Valparaiso, CHILE

**Keywords:** Biological Sciences, Developmental Neuroscience, Developmental Biology

## Abstract

Eclosion hormone (EH) was originally identified as a brain-derived hormone capable of inducing the behavioral sequences required for molting across insect species. However, its role in this process (called ecdysis) has since been confounded by discrepancies in the effects of genetic and cellular manipulations of EH function in *Drosophila*. Although knock-out of the *Eh* gene results in severe ecdysis-associated deficits accompanied by nearly complete larval lethality, ablation of the only neurons known to express EH (i.e. V_m_ neurons) is only partially lethal and surviving adults emerge, albeit abnormally. Using new tools for sensitively detecting *Eh* gene expression, we show that EH is more widely expressed than previously thought, both within the nervous system and in somatic tissues, including trachea. Ablating all *Eh*-expressing cells has effects that closely match those of *Eh* gene knock-out; developmentally suppressing them severely disrupts eclosion. Our results thus clarify and extend the scope of EH action.

## Highlights

• *Eh* is expressed in non-neuronal peripheral tissues including trachea
• *Eh* expression is found in neurons other than the V_m_ neurons in pharate adults
• Non-V_m_ expression is essential for eclosion
• Non-neuronal *Eh* expression is required for normal larval ecdysis

## Graphical Abstract

**Figure:**
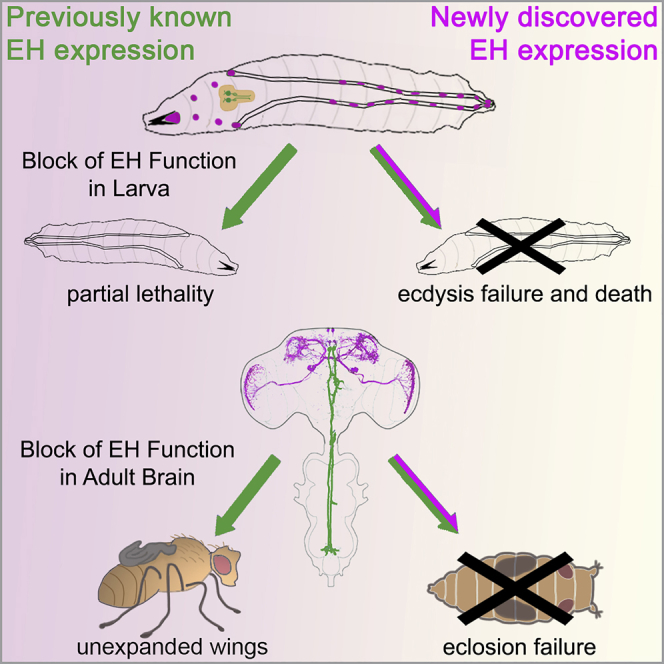

## Introduction

An essential feature of insect development is the periodic replacement of the exoskeleton, which not only protects the body but also lines the airways (i.e. trachea) and portions of the gut (Truman, 2005, White and Ewer, 2014, Zitnan and Adams, 2012). The process of replacing it, called ecdysis, is hormonally mediated and requires the execution of a behavioral program called an ecdysis sequence. EH was the first hormone shown to be instrumental in initiating ecdysis sequences in insects (Truman and Riddiford, 1970, Truman et al., 1981). Its neural origin was originally demonstrated by brain transplantation studies , and subsequent analysis by mRNA *in situ* hybridization and immunohistochemistry identified its principal site of release to be two to four large neurosecretory cells (i.e. V_m_ neurons) named for their ventromedial disposition in the brains of most insects (Truman and Copenhaver, 1989, Horodyski et al., 1989). The discovery of EH was followed by the identification of a second hormone that was of non-neural origin but was likewise capable of potently eliciting insect ecdysis sequences (Park et al., 1999, Roller et al., 2010, Zitnan et al., 1996). This hormone, called ecdysis triggering hormone (ETH), is released into the hemolymph from tracheal-associated cells (i.e. Inka cells) and acts at multiple sites in the brain, including the V_m_ neurons (Diao et al., 2016, Kim et al., 2006a, Kim et al., 2006b). EH reciprocally targets the Inka cells, and strong positive feedback between ETH and EH signaling insures cooperative release of both hormones at the time of ecdysis (Ewer et al., 1997, Kingan et al., 1997).

This interdependence of EH and ETH action has complicated efforts to tease apart the individual functions of the two hormones. An additional confound has been the ambiguous effects of genetic versus cellular manipulations of EH function. In *Drosophila*, the only known sources of EH are the two V_m_ neurons (Horodyski et al., 1993). Ablating these neurons causes aberrant ETH release from the Inka cells, but larvae lacking V_m_ neurons exhibit only minor behavioral deficits at ecdysis (Clark et al., 2004). Approximately two-thirds of such larvae die from failures in tracheal air filling, a process that normally precedes cuticle shedding, but the remainder survive to adulthood and, perplexingly, exhibit only non-lethal deficits in eclosion, such as wing expansion failure (McNabb et al., 1997). These results are in striking contrast to the effects of knocking out the *Eh* gene: 90% of *Eh* null mutants die as larvae and none survive to adulthood (Kruger et al., 2015). No detectable release of ETH from the Inka cells is seen in these animals, and those surviving to the second larval molt fail to execute the first phase of the ecdysis sequence (i.e. pre-ecdysis), a deficit that is not rescued by injection of ETH.

The substantial discrepancies in the effects of EH gene knock-out and V_m_ neuron ablation strongly suggest additional sources of EH in the fly brain outside of the V_m_ neurons. To identify other possible sources of EH, we have applied the Trojan exon method (Diao et al., 2015), which permits sensitive detection and functional manipulation of cells expressing a gene of interest. Using this method, together with a newly generated anti-EH antibody, we have identified novel EH-expressing neurons, which together with the V_m_ neurons govern adult ecdysis behavior. Surprisingly, the non-V_m_ neurons are absent until late in larval development, but we find that the Eh gene is expressed in larvae by tracheal and other somatic cells. Ablation of these cells disrupts ecdysis and, like *Eh* gene knock-out, is larval lethal. Our results resolve discrepancies in EH action and suggest a broader role for trachea in ecdysis than previously appreciated.

## Results

### EH Is Expressed in Neurons Other Than the V_m_ Neurons of *Drosophila*

**Figure 1:**
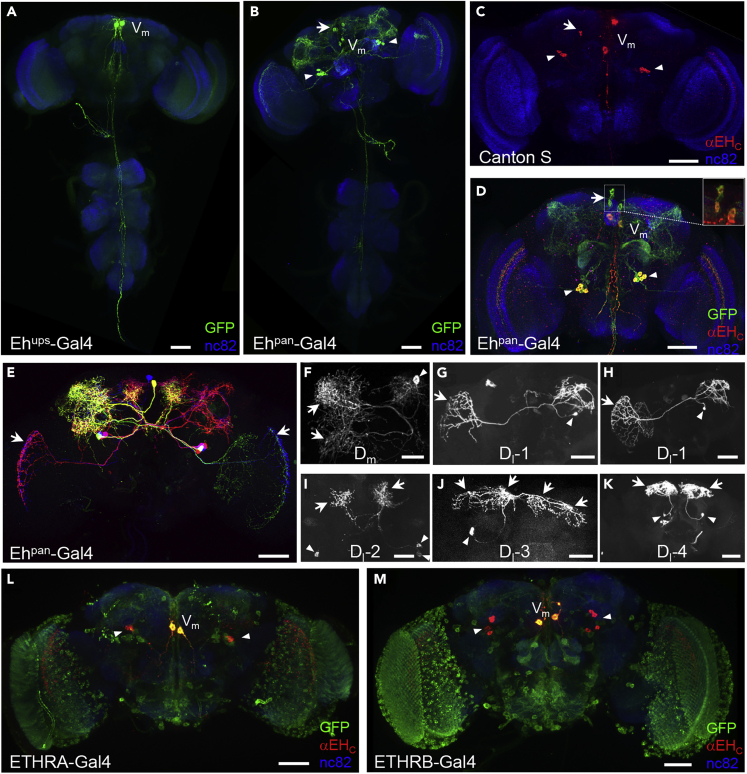
EH Is Expressed in Neurons Other than the V_m_ Neurons in Pharate Adults (A) *Eh*^*ups*^*-Gal4* drives UAS-*CD4::tdGFP* expression (green) only in the two ventromedial (i.e. V_m_) neurons of the central brain in a fluorescence confocal image of a pharate adult CNS wholemount. Blue, neuropil counterstained with nc82 antibody. Scale bar in all images: 50 μm. See also Figure S1A. (B) *Eh*^*pan*^*-Gal4* drives UAS-*CD4::tdGFP* expression (green) in two groups of neurons in addition to the V_m_ neurons: the D_l_ (arrowheads) and D_m_ (arrow) groups. The dorsal disposition of these neurons is relative to the neuraxis (i.e. n-dorsal, following the nomenclature of Ito et al., 2014). See also Figures S1A and S1B. (C) Anti-EH immunostaining of pharate adults with the αEH_C_ antibody (red) reveals neurons in addition to the V_m_s in the CNS wholemount of a wild-type, Canton-S animal. The positions of these cell groups (arrowheads and arrow) are similar to those of the D_l_ and D_m_ groups in (B). See also Figures S1D and S1E. (D) *Eh*^*pan*^*-Gal4*-driven expression of UAS-*CD4::tdGFP* (green) overlaps with anti-EH immunostaining (αEH_C_, red) in the D_l_ (arrowheads) and D_m_ (arrow) groups. Inset: double-labeling of D_m_ neurons (with green and red channel gains adjusted). Immunostaining of these neurons was generally weak and inconsistent. (E) Multicolor FlipOut (MCFO) labeling of neurons in the *Eh*^*pan*^*-Gal4* expression pattern. Individual EH-expressing neurons are stochastically labeled in different colors depending on the fluorescent markers they express. (F) A typical D_m_ neuron labeled by MCFO. Somata (arrowhead) of all D_m_ neurons are located near the midline at the level of the Antler and typically innervate the Superior Lateral Protocerebrum. (G–K) Four types of D_l_ neurons were distinguished by MCFO. Somata of all types were at the level of the superior posterior slope; arrows indicate projections. Type 1 neurons (G, H) were distinguished by their innervation of the medulla in the contralateral optic lobe. The scope of optic lobe innervation varied among type 1 neurons. The axons of type 2 neurons (I) crossed the inferior bridge and ramified in the Superior Medial Protocerebrum (SMP). Type 3 (J) and type 4 (K) neurons also arborize in the SMP. The latter often does so only ipsilaterally, whereas the former projects bilaterally and also innervates the Superior Lateral Protocerebrum. (L–M) Expression of the two subtypes of ETH receptor, revealed by expression of *ETHRA-Gal4*(J) and *ETHRB-Gal4*(K), respectively, overlaps with expression of EH (αEH_C_, red) in the V_m_ neurons but not D_l_ neurons. Green, UAS-*CD4::tdGFP*; blue, nc82.

Previous characterization of EH-expressing cells in the *Drosophila* central nervous system (CNS) have relied on *Eh*^*ups*^*-Gal4*, a promoter fusion line that selectively labels the V_m_ neurons (Figures 1A and S1A) (McNabb et al., 1997). To drive Gal4 expression, this line uses 2.4 kb of DNA located directly upstream of the *Eh* coding sequence, which may lack the full complement of enhancer domains responsible for native EH expression. To more faithfully capture the native expression pattern, we generated modified Trojan Gal4-and p65AD-expressing lines with insertions into the third intron of the *Eh* gene (Figure S1A). In pharate adults, these lines drive expression of a GFP reporter not only in the V_m_ neurons but also in two dorsal groups of neurons (“n-dorsal” following the nomenclature of Ito et al., 2014) in the central brain (Figures 1B, S1B, and S1C; note that all fly lines and all genotypes for crosses used in this study are listed in Tables S1 and S2, respectively). One of these groups consists of 6–7 (6.4 ± 1.3, n = 6) closely clustered neurons laterally disposed in each brain hemisphere with cell bodies near the superior posterior slope. We call these the dorsolateral (D_l_) neurons (Figure 1B, arrowheads). The second group consists of approximately three pairs of neurons (6.29 total ± 0.76, n = 7) clustered around the midline at the level of the Antler, which we call the dorsomedial (D_m_) neurons (Figure 1B, arrow).

To verify expression of EH in these neurons, we generated a high-affinity antibody against the C-terminus of the *Drosophila* EH protein. In CNS wholemount preparations from wild-type animals, this antibody (αEH_C_) recognized not only the V_m_ neurons but also groups of cells similar in location to those of the D_l_ and D_m_ clusters (Figure 1C). To demonstrate the specificity of the antibody, we immunostained CNS preparations from *Eh* null mutant pharate adults in which EH was misexpressed in the peritracheal Inka cells (Park et al., 2002). Such misexpression has been previously shown to rescue larval ecdysis deficits associated with *Eh* gene knock-out (Kruger et al., 2015), and we find that the CNS of such animals is devoid of immunostaining, validating the specificity of antibody labeling (Figure S1D). Older adults lack anti-EH_C_immunoreactivity in the V_m_ neurons, which undergo apoptosis after eclosion (data not shown), but they retain it in the D_l_ neurons (Figure S1E). Double-labeling of the brains of *Eh*^*pan*^*>mCD8-GFP* animals confirmed that the novel EH-immunoreactive neurons corresponded to those of the D_l_ and D_m_ clusters (Figure 1D). Within the D_l_ cluster, approximately half of the six neurons in each hemisphere (3.3 ± 1.4; n = 6) were consistently immunoreactive in the preparations examined, whereas neurons within the D_m_ cluster were weakly and less consistently double-labeled (Figure 1D, inset). Indeed, some genotypes, such as *w*^*1118*^ mutants, typically lacked αEH_C_ immunostaining in the D_m_ neurons entirely, suggesting that EH expression in this cell type is dispensable.

To characterize the anatomy of the novel EH-expressing neurons, we labeled them individually using the MultiColor FlpOut (MCFO) technique (Nern et al., 2015) with the *Eh*^*pan*^*-Gal4* line (Figures 1E–1K). We find that both types of neurons in the D_m_ cluster have similar morphologies (Figure 1E, yellow neuron; Figure 1F) and send projections to the lateral horn and posterior lateral protocerebrum. Cell types of the D_l_ cluster are more diverse with evidence for at least four major types (Figures 1G–1K). Type 1 neurons send axonal projections across the inferior bridge to the contralateral optic lobe where their terminals form putative EH release sites that decorate layers M7 and/or M8 of the medulla to varying degrees (Figures 1E, 1G, and 1H, arrows). These neurons, of which there are at least two per cluster, also have an ipsilateral (possibly dendritic) projection to the Lateral Horn. The other three types of D_l_ neurons send prominent projections to one or both sides of the Superior Medial Protocerebrum but differ in the anatomy of their arbors and/or site of midline crossing (Figures 1I–1K). Unlike the V_m_ neurons, which are targets of ETH (Diao et al., 2016, Kim et al., 2006b), the D_l_ neurons do not express either subtype of the ETH receptor, ETHRA or ETHRB (Figures 1L and 1M). Absence of αEH_C_ immunoreactivity in the D_m_ neurons in preparations expressing reporters driven by either ETHR Gal4 line precluded a definitive conclusion about EH expression in these cells. However, previous observations that ablation of the V_m_ neurons alone using *Eh*^*ups*^*-Gal4* eliminates sensitivity to injected ETH (Clark et al., 2004, McNabb et al., 1997) is consistent with the conclusion that ETHR expression is restricted to only the V_m_ neurons.

### EH-Expressing Cells Distinct from the V_m_ Neurons Are Required for Ecdysis

We used two copies of the inwardly rectifying K^+^ channel, UAS-*Kir2.1*, to electrically silence either the full complement of EH-expressing neurons or V_m_ neurons alone using the *Eh*^*pan*^- and *Eh*^*ups*^*-Gal4* drivers, respectively (Table 1). Silencing the V_m_ neurons alone substantially reduced larval viability, with only 30% of animals surviving to the pupal stage. Of the survivors, however, 90% successfully eclosed as adults. These results are similar to what has previously been observed with cell-type specific ablation of the V_m_ neurons (McNabb et al., 1997). In contrast, no larvae survived when all EH-expressing neurons were suppressed, and many dying animals exhibited deficits in cuticle shedding and tracheal filling at early larval molts. The penetrance of the lethality suggests that the cells targeted by *Eh*^*pan*^*-Gal4*, as opposed to *Eh*^*ups*^*-Gal4*, are likely to represent most, if not all, of the sources of secreted EH. These differing effects also argue strongly for the functional importance of the non-V_m_ population of EH-expressing cells.

**Table 1:** Suppression of *Eh*-Expressing Cells Is Lethal at the Larval Stage

Parental Genotypes	Embryos	Animals that Pupariate (%)	Pupae that Eclose (%)	Cumulative Survival to Adult (%)	Adults with Wings Expanded (%)
*w*^*1118*^ x 2xUAS-*Kir2.1*	380	90.8	98.3	89.2	100
*Eh*^*ups*^ x 2xUAS-*Kir2.1*	570	29.6	89.9	26.7	96.1
*Eh*^*pan*^ x 2xUAS-*Kir2.1*	628	0.2	0	0	NA

**Figure 2:**
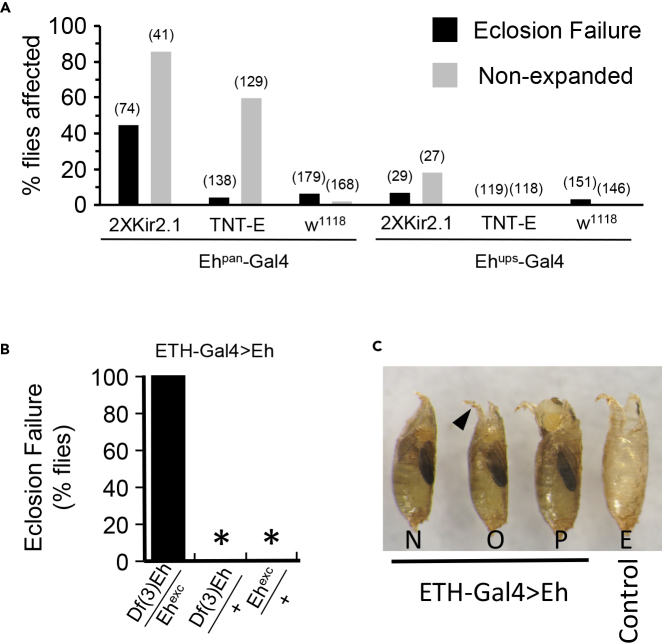
Non-V_m_ Neurons Are Required for Normal Adult Ecdysis (A) Suppression of neuronal function mediated by *Eh*^*pan*^*-Gal4* causes more penetrant adult ecdysis deficits than suppression mediated by *Eh*^*ups*^*-Gal4*. 2XUAS-*Kir2.1* and UAS-*TNT-E* were used to suppress neuronal excitability and synaptic transmission, respectively, and animals were assayed for eclosion (black bars) and wing expansion (gray bars) failure. For *Eh*^*pan*^*-Gal4*, suppression was limited to the adult stage using *tubP-Gal80ts*. Progeny of Gal4 control crosses to *w*^*1118*^ flies were assayed in parallel. N for each phenotype in parentheses. (B) *Eh* null mutants (*Df(3)Eh/Eh*^*exc*^) expressing a UAS-*Eh* rescue construct in the Inka cells using *ETH-Gal4* survive to adulthood but then fail to eclose. Two control genotypes hemizygous for the *Eh* gene eclose successfully (∗, 0%). (C) Eclosion deficits of the *Df(3)Eh/Eh*^*exc*^ mutants rescued by *ETH-Gal4*>UAS-*Eh* expression included complete failure to eclose (N), eclosion failure with operculum opening (O), and partial eclosion (P). Control animals eclosed (E), leaving an empty puparium.

To examine the adult-specific effects of inhibiting EH-expressing neurons, we used the temperature-sensitive blocker of GAL4, GAL80ts (McGuire et al., 2003), to limit UAS-*Kir2.1* activity to the period of pupal development. Using *Eh*^*pan*^*-Gal4*, such silencing caused profound deficits in adult ecdysis, with nearly half of animals failing to eclose (Figure 2A). Of those that did, 85% had substantial deficits in wing expansion, a process that completes the adult ecdysis sequence and which has previously been shown to be disrupted by V_m_ neuron ablation (McNabb et al., 1997). A second suppressor of neuronal activity, UAS-*TNT* (Sweeney et al., 1995), also substantially blocked wing expansion when expressed in all EH-expressing cells, but had only a minor effect on eclosion. Minor effects on both eclosion and wing expansion were also observed when the V_m_ neurons alone were suppressed using *Eh*^*ups*^*-Gal4*, again consistent with previous reports (McNabb and Truman, 2008).

The substantial eclosion deficits seen when all EH-expressing neurons are suppressed, versus only the V_m_ neurons, strongly suggests that cells other than the V_m_ neurons function to support the process of eclosion. To more directly assess the effects of EH loss-of-function in the brain, we took advantage of the previously reported observation that *Eh* null mutants ectopically expressing a UAS-*Eh* transgene in the peritracheal Inka cells execute relatively normal larval ecdysis (Kruger et al., 2015). Indeed, we find that such animals are not only viable through larval life but also develop without overt abnormalities as pupae. However, almost none (1/297) eclose, although 53% (156/297) do so partially (Figures 2B and 2C). These animals successfully open the operculum of their puparium but then either fail to emerge (Figure 2C, “O,” arrowhead) or only partially emerge (Figure 2C, “P”). All other animals completely failed to eclose, in contrast to control animals hemizygous for the *Eh* gene, which all eclosed normally.

Operculum opening requires expansion of the ptilinum by rhythmic contraction of thoracic muscles prior to eclosion (Miyan, 1989). The observation that many non-eclosing animals opened their opercula and that some partially emerged indicates that they initiated the ecdysis sequence. Video observation confirmed that, in general, *Eh* null mutants rescued by ectopic EH expression in the Inka cells repeatedly inflated their ptilina and also displayed abdominal contractions (Video S1, right). Abdominal contractions, however, were sporadic, and the coordinated, rhythmic peristalses that wild-type animals use to exit the puparium were not observed (Video S1, left). In addition, these animals also often appeared to initiate ptilinum expansion prior to molting fluid resorption.

Video S1.Eclosion Failure in the Absence of Brain EH Expression, Related to Figure 2 Right, an *Eh* null mutant (*Df(3)Eh/Eh*^*exc*^) developmentally rescued by expression of a UAS-*Eh* transgene under the control of the *ETH-Gal4* driver. Left, a Canton-S, wildtype control animal. Video speed: 8X.

### EH Is Expressed outside of the Nervous System

**Figure 3:**
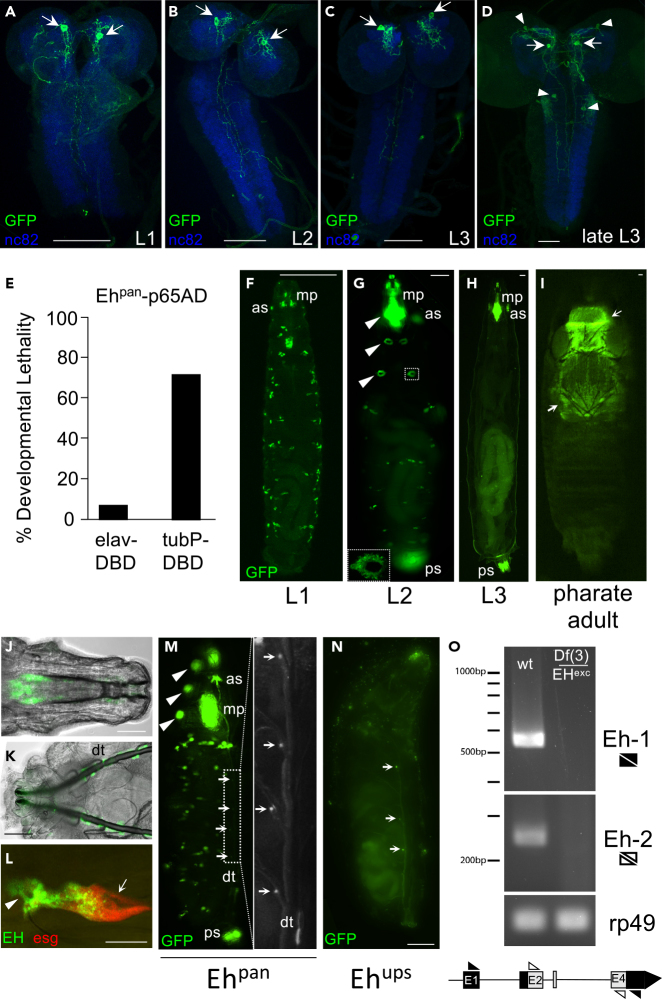
EH Is Expressed Outside the Nervous System in Larvae (A–D) Confocal micrographs of CNS wholemounts from L1 (A), L2 (B), early L3 (C), and wandering L3 (D) larvae showing the expression pattern of *Eh*^*pan*^*-Gal4* driving a UAS-*CD4::tdGFP* reporter (green). At all stages, the V_m_ neurons (arrows) are labeled, but only late L3 larvae express reporter in non-V_m_ neurons (arrowheads). Blue, anti-nc82 immunostaining of neuropil. Scale bar: 50 μm. (E) Suppression of all EH-expressing cells (*tubP-Gal4DBD*) results in greater developmental lethality than suppression of all EH-expressing neurons (*elav-Gal4DBD*). Flies bearing an *Eh*^*pan*^*-p65AD* hemidriver over a TM3, *Sb* balancer were crossed to flies bearing the indicated *Gal4DBD* hemidrivers and UAS-*Kir2.1*. Bar graph shows the eclosing progeny that received the *Eh*^*pan*^*-65AD* hemidriver as a percentage of those that received the TM3, *Sb* balancer. See also Figures S1A and S1C. (F–I) Fluorescence micrographs showing somatic expression of *Eh*^*pan*^*>CD4::tdGFP* (green) in living (F) L1, (G) L2, and (H) L3 larvae, as well as (I) pharate adult. All larvae are shown from the ventral side. The dorsal side of the adult is shown. Identified labeled tissues include the following: mp, mouth parts; as, anterior spiracles; and ps, posterior spiracles. Arrowheads, labeled ring structures surrounding Keilin's organs (see text and inset in panel G). Arrows, labeling of presumptive air sacs of the thorax and head in the pharate adult (ptilinum extended). Scale bars: 100 μm. (J–L) Somatic expression of *Eh*^*pan*^*>CD4::tdGFP* (green) in larvae that have just completed L1-L2 ecdysis. Micrographs show labeling of structures associated with (J) the cephaloskeleton, (K) the dorsal tracheal trunks, and (L) *Eh*^*pan*^*-Gal4* expression (green) in a group of cells surrounding Keilin's organ (arrowhead) in an L2 larva shortly after completing ecdysis. An *esg* promoter-reporter (red) labels leg disc (arrow) as well as co-labeling EH expressing cells. Scale bar: 50 μm. (M) Somatic expression in a lateral view of an *Eh*^*pan*^*>CD4::tdGFP* (green) larva at the same stage as in (J–L) showing expression surrounding Keilin's organs (arrowheads), the mouthparts (mp), anterior (as) and posterior (ps) spiracles, and various other cells along the body wall. Inset shows cells located on the segmental tracheal branches near the junction with the tracheal trunk. dt, dorsal tracheal trunk cells. For scale bar see (N). (N) Somatic expression of *Eh*^*ups*^*>CD4::tdGFP* (green) in a larva at the same stage as animal in (M) and imaged from the lateral side at equivalent camera settings. Arrows indicate cells allied with segmental branches of the tracheal trunks. Scale bar: 100 μm. (O) RT-PCR amplifies *Eh*-specific sequences from tracheal RNA prepared from L1/L2 larvae of wildtype, but not *Eh* null mutant (*Df(3)Eh/Eh*^*exc*^) animals. The two primer pairs used for amplification are indicated schematically. Spaces between bands indicate where the gel was cut to conserve space.

Taken together, the above results strongly implicate a role for the non-V_m_ population of EH-secreting neurons in adult ecdysis. These neurons were also obvious candidates for the profound larval lethality observed upon silencing all EH-expressing cells. However, examination of both the *Eh*^*pan*^*-Gal4* expression pattern (Figures 3A–3D) and anti-EH_C_ immunoreactivity (data not shown) in the CNS at different larval stages showed that only the V_m_ neurons were labeled before the late third larval instar and therefore after the stage at which lethality is seen in *Eh* null mutants. Wandering L3 larvae do exhibit Gal4 expression in two additional pairs of non-V_m_ neurons (Figure 3D, arrowheads), both of which exhibit only weak and transient anti-EH_C_ antibody staining (data not shown). Driving UAS-*Kir2.1* in EH-expressing neurons using an *Eh*^*pan*^*-p65AD*∩*elav-Gal4DBD* Split-GAL4 driver also resulted in considerably less developmental lethality than ubiquitous expression of UAS-*Kir2.1* achieved using *Eh*^*pan*^*-p65AD* with a *tubulin* promoter-driven *tubP-Gal4DBD* (Figures 3E, S1A, and S1C). These results suggest that a non-neuronal source of EH is responsible for the lethality observed at the larval stage, a conclusion that may explain the ability of EH ectopically expressed in the Inka cells to rescue larval ecdysis in *Eh* null mutants.

We used the *Eh*^*pan*^*-Gal4* driver to examine somatic expression of a UAS-*CD4::tdGFP* reporter in larvae. We observed expression in multiple tissues at all three larval instars (Figures 3F–3H). The distribution of signal appeared to become more restricted with increasing larval age although post-larval somatic expression was also evident in pharate adults in presumptive thoracic and head air sacs (Figure 3I, Whitten, 1957). The latter structures are part of the tracheal system as are the anterior and posterior spiracles (as and ps, respectively in Figures 3F–3H), two structures consistently labeled in larvae at all stages along with tissues associated with the mouthparts (mp, Figures 3F–3H and3J). Expression, in general, was dynamic, particularly at L1, where the distribution and intensity of labeling was highest in animals undergoing L1-L2 ecdysis (Figures 3J–3M). In addition to spiracle and mouthpart labeling, the expression pattern at this time typically included epithelial cells of the dorsal tracheal trunks (Figures 3K and 3M; dt), numerous superficial cells along the ventral and lateral body wall, and cells in segments T1–T3 surrounding three pairs of larval sensory structures associated with the leg imaginal discs, known as Keilin's organs (Figures 3G, inset, 3L and 3M, arrowheads Lakes-Harlan et al., 1991, McKay et al., 2009). Also weakly labeled were cells on the segmental branches of the dorsal tracheal trunks (Figure 3M, inset; arrowheads). Interestingly, these cells were the only consistent site of labeling of the *Eh*^*ups*^*-Gal4* driver, the somatic expression of which was, in general, weak and sparse (Figure 3N; arrows). These cells are intriguing because of their proximity to the ETH-expressing Inka cells, which are located at the base of the tracheal branches along the dorsal trunks. They thus represent a possible—and strictly peripheral—site of interaction between the EH and ETH signaling systems. More work will be required to investigate this possibility, but the presence of these cells in the expression patterns of both *Eh*^*pan*^- and *Eh*^*ups*^*-Gal4* drivers indicates that they are unlikely to account for the phenotypic differences observed in manipulations of EH signaling performed with these two drivers.

Our efforts to confirm the expression of EH at somatic sites by immunostaining were unsuccessful—perhaps due to low hormone levels—but RT-PCR revealed the presence of *Eh* message in RNA preparations made from tracheal tissues of L1 and L2 larvae (Figure 3O). Two primer pairs directed against unique sequences in the coding or non-coding regions of the *Eh* gene both yielded bands of the expected size and sequence when amplified by RT-PCR. These bands were, however, missing from tracheal RNA preparations made in parallel from *Eh* null mutants. The relative levels of *Eh* mRNA contributed to our RNA preparations by the various tracheal and tracheal-associated cell types remain to be determined, but the prevalence of *Eh* expression in tissues affiliated with the trachea suggests a role for these tissues in EH signaling that extends beyond the ETH-secreting Inka cells.

### Non-neuronal Expression of EH Is Critical for Larval Ecdysis

**Figure 4:**
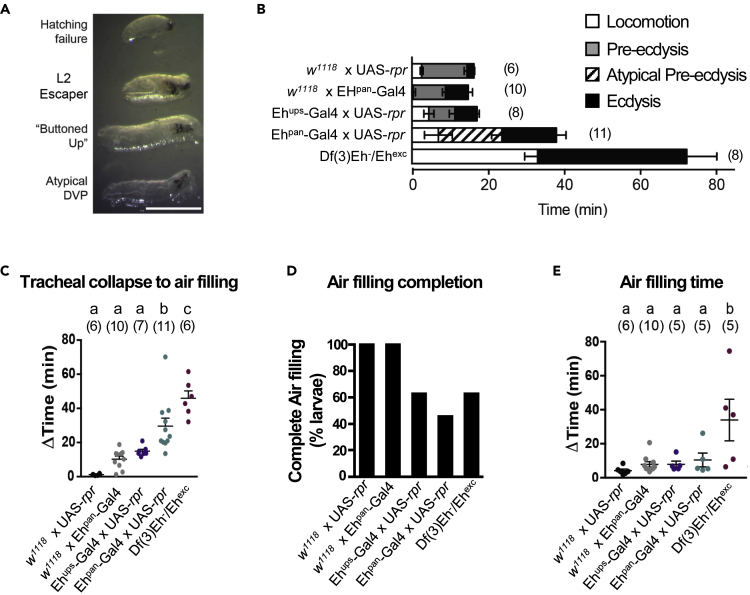
Ablating EH-Expressing Cells Causes Deficits Similar to Those Seen in *Eh* Null Mutants (A) Lethal phenotypes observed in *Eh*^*pan*^*>rpr* animals. A few animals failed to hatch or died as older L2 larvae, but most died shortly after completion of the L1-L2 ecdysis sequence without shedding their cuticles (i.e. “buttoned-up” phenotype) or other defects. DVP: double vertical plates, a marker for imminent ecdysis. Scale bar: 500 μm. (B) Behavioral analysis of animals undergoing L1-L2 ecdysis. Bar graphs indicate the presence, normality, and duration of ecdysis sequence phases in experimental and control animals of the indicated genotypes. Time zero corresponds to the time of tracheal collapse, a marker of ecdysis onset. Error bars indicate standard error of the mean and (n) indicates the number of animals analyzed. (C–E) Tracheal filling at L1-L2 ecdysis in animals of the same genotypes analyzed for behavior in (B). The parameters measured were (C) the time from collapse of the old trachea to the filling of the new ones, (D) the percentage of animals that completely filled their trachea with air, and (E) the time from the beginning to the end of tracheal air filling. (E) excludes animals from (D) that failed to fill their trachea, and three animals that never began air filling were excluded from (C). Data were analyzed by one-way ANOVA and Tukey multiple comparison. Statistically significant differences are represented by different letters.

Further investigation will also be required to determine the exact identities of the tracheal and other somatic cell types in which *Eh*^*pan*^*-Gal4* is expressed. The strong effects of UAS-*Kir2.1* expression in these cells, however, suggest that a depolarization-dependent process is being suppressed—e.g. release of EH from a novel class of excitable secretory cells. Given the unexpected nature of our observation, we also generated loss-of-function phenotypes using a more conventional manipulation that has routinely been applied in studies of V_m_ neuron function. We used *Eh*^*pan*^*-Gal4* to genetically ablate all EH-expressing cells using *UAS-rpr* (Baker et al., 1999, Clark et al., 2004, McNabb et al., 1997). Larval lethality was again complete, apart from a few animals that failed to hatch, and almost all animals died shortly after the first larval ecdysis with various defects (Figure 4A). None survived past L2.

To assess the behavioral and physiological effects of ablation, we video-recorded 11 animals at the time of L1-L2 ecdysis and assayed their behavior and tracheal air filling (Figures 4B–4E). Similar to *Eh* null mutants, *Eh*^*pan*^*>rpr* animals successfully executed the motor patterns associated with ecdysis but were slow to initiate and complete this behavior compared with control or *Eh*^*ups*^*>rpr* animals (Figure 4B). Although *Eh*^*pan*^*>rpr* larvae displayed pre-ecdysis behavior, it was aberrantly executed and nearly two-thirds (7/11) of these animals failed to shed their cuticles and mouth hooks and resembled the “buttoned-up” phenotype described for *ETH* null mutants (Park et al., 2002). All 11 died shortly after ecdysis. In contrast, only two of eight *Eh* null mutants observed failed to shed their cuticles, and all *Eh*^*ups*^*>rpr* animals did so after successfully executing ecdysis. *Eh*^*ups*^*>rpr* larvae did exhibit some deficits in tracheal air filling, as previously reported (Clark et al., 2004, McNabb et al., 1997), but these were generally less severe than those of *Eh*^*pan*^*>rpr* and *Eh* null mutant animals (Figures 4C–4E). Overall, ablating all EH-expressing cells produced effects much more similar to those of eliminating the *Eh* gene than those produced by ablating only the V_m_ neurons.

## Discussion

The results presented here challenge the long-held assumption that EH is a strictly brain-derived hormone in insects, secreted solely by pairs of V_m_ cells in the central brain. In *Drosophila*, we demonstrate that *Eh* is expressed in neurons other than the V_m_ neurons at the adult stage and in somatic tissues at all stages. Suppression of function of the full complement of EH-expressing cells both at the larval and the adult stages produces ecdysis deficits considerably more severe than those of V_m_ loss-of-function alone. The non-V_m_ cells thus clearly play important roles in ecdysis, and overall, our results indicate that EH signaling is more broadly distributed—and likely more diversely regulated—than has been hitherto appreciated.

The primary regulator of EH secretion according to current models is ETH, which is secreted from the epitracheal Inka cells and potently facilitates EH release from the V_m_ neurons (Ewer et al., 1997, Kingan et al., 1997). Our finding that the D_l_ neurons do not express either isoform of the ETHR indicates that EH release from these cells must be governed by some other mechanism. Whether the D_m_ and somatic cells are also regulated by mechanisms other than ETH remains to be determined, but their sheer variety suggests that they likely serve different functions. Indeed, the D_l_ neurons also appear to have functions beyond ecdysis in that they persist into adulthood. The morphology of the type 1 D_l_ neurons, with presumptive EH release sites in the medulla, suggests that they may modulate visual processing. Interestingly, acute exposure to light gates eclosion in flies (Engelmann and Honegger, 1966), an effect has been attributed to light simultaneously stimulating EH release from the V_m_ neurons and disinhibiting the eclosion motor program (McNabb and Truman, 2008). It is possible that the D_l_ neurons may sensitize visual pathways that promote these effects. An important goal of future work will be to investigate possible interactions and synergies between the D_l_ and V_m_ neurons and other cell types involved in EH signaling.

Another goal of future work will be to determine the function of EH expression in somatic cells. The expansion of this expression at the time of larval ecdysis is consistent with a role in that process and may relate to the refilling of the new trachea with air (Baker et al., 1999, Clark et al., 2004, McNabb et al., 1997). Replacement of the trachea of the previous developmental stage with larger trachea is required to accommodate the metabolic needs and increased oxygen demands of the growing animal (Harrison et al., 2018, Kivela et al., 2016), and ETH, as well as EH, has been implicated in this process (Park et al., 2002). Somatically expressed EH may directly promote tracheal air filling and/or indirectly promote it via interactions with the epitracheal Inka cells, which express ETH. Given that limitations in tracheal size participate in initiating molting (Callier and Nijhout, 2011), and that both EH and ETH are expressed by tracheal-associated cells, it is interesting to speculate that the trachea may act as a convergence point for organizing ecdysis-related events.

It will also be interesting to examine to what extent our findings in *Drosophila* generalize. Prior to the discovery of the V_m_ neurons, other neurons in the hawkmoth, *Manduca* *sexta*, were proposed to be sources of EH (Copenhaver and Truman, 1986). However, the function of these neurons has not been further characterized, and EH expression outside of the nervous system remains to be examined in other insects. It is worth noting that our identification of novel *Eh*-expressing cells in *Drosophila* was made possible by our use of the Trojan exon method, which is capable of capturing all of the regulatory elements driving *Eh* gene expression (Diao et al., 2015). An *Eh-T2A-LexA* line published—but not commented on—by Deng et al. (2019) was similarly designed to co-opt all regulatory information of the native *Eh* gene and appears to have a CNS expression pattern very similar to that of *Eh*^*pan*^*-Gal4*, including expression in non-V_m_ neurons.

Critical regulatory elements for *Eh* expression are evidently missing from the original *Eh*^*ups*^*-Gal4* driver, which expresses in few somatic cells and in only the V_m_ neurons in the CNS. Interestingly, a driver line in the Janelia FlyLight collection (*R60F12-Gal4*; https://www.janelia.org/project-team/flylight) in which Gal4 expression is driven by a 1,293 bp genomic fragment comprising the first intron of the *Eh* gene does not appreciably label the V_m_ neurons but does label numerous other cells, including groups with striking similarity to the D_l_ and D_m_ neurons. This intronic enhancer thus appears to contain complementary information for neuronal *Eh* expression to that contained in the 2.4 kb of upstream DNA used to make the *Eh*^*ups*^*-Gal4* driver. The regulatory elements that determine *Eh* expression in somatic cells have yet to be identified. In general, however, the new tools introduced here should help to more fully characterize the regulation, timing, and extent of EH expression in *Drosophila* and to facilitate a more thorough-going investigation of the mechanisms by which EH acts.

### Limitations of the Study

Although the RT-PCR evidence presented here confirms *Eh* gene expression in cells associated with the trachea, *Eh* expression in other somatic cell types remains to be demonstrated. Also, although the deficits caused by ablation of all identified *Eh*-expressing cells strongly resemble those caused by *Eh* gene knock-out, they do not precisely phenocopy them. In particular, animals lacking the *Eh* gene typically exhibit more severe tracheal air-filling deficits than those lacking the EH-expressing cells. This is an unexpected finding in that disrupting cellular function should affect more processes than simply eliminating EH activity. These discrepancies await to be resolved. Finally, our evidence that neuronal expression of *Eh* is required for eclosion rests on the assumption that secretion of mis-expressed EH by the Inka cells at the time of eclosion mimics EH secretion by other somatic tissues that might normally express this hormone (such as the air sacs). It is possible that this is not the case or that the timing of EH secretion by the Inka cells disrupts ETH release at adult ecdysis in some way that it does not at earlier stages. These caveats will have to be addressed using other methods.

## Resource Availability

### Lead Contact

Further information and requests for resources and reagents should be directed to and will be fulfilled by the Lead Contact, Benjamin H. White (benjaminwhite@mail.nih.gov).

### Materials Availability

All reagents generated in this study are available from the Lead Contact without restriction.

### Data and Code Availability

The datasets supporting the current study are available from the corresponding author on request.

## Methods

All methods can be found in the accompanying Transparent Methods supplemental file.

## Supplemental Information

Document S1. Transparent Methods, Figures S1, and Table S1

## References

[bib1] Baker J.D., McNabb S.L., Truman J.W. (1999). The hormonal coordination of behavior and physiology at adult ecdysis in Drosophila melanogaster. J. Exp. Biol..

[bib2] Callier V., Nijhout H.F. (2011). Control of body size by oxygen supply reveals size-dependent and size-independent mechanisms of molting and metamorphosis. Proc. Natl. Acad. Sci. U S A.

[bib3] Clark A.C., Del Campo M.L., Ewer J. (2004). Neuroendocrine control of larval ecdysis behavior in Drosophila: complex regulation by partially redundant neuropeptides. J. Neurosci..

[bib4] Copenhaver P.F., Truman J.W. (1986). Identification of the cerebral neurosecretory-cells that contain eclosion hormone in the moth manduca-sexta. J. Neurosci..

[bib5] Deng B.W., Li Q., Liu X.X., Cao Y., Li B.F., Qian Y.J., Xu R., Mao R.B., Zhou E.X., Zhang W.X. (2019). Chemoconnectomics: mapping chemical transmission in Drosophila. Neuron.

[bib6] Diao F., Ironfield H., Luan H., Diao F., Shropshire W.C., Ewer J., Marr E., Potter C.J., Landgraf M., White B.H. (2015). Plug-and-play genetic access to drosophila cell types using exchangeable exon cassettes. Cell Rep..

[bib7] Diao F.C., Mena W., Shi J., Park D., Diao F.Q., Taghert P., Ewer J., White B.H. (2016). The splice isoforms of the Drosophila ecdysis triggering hormone receptor have developmentally distinct roles. Genetics.

[bib8] Engelmann W., Honegger H.W. (1966). Tagesperiodische schlupfrhythmik einer augenlosen Drosophila melanogaster-mutante. Naturwissenschaften.

[bib9] Ewer J., Gammie S.C., Truman J.W. (1997). Control of insect ecdysis by a positive-feedback endocrine system: roles of eclosion hormone and ecdysis triggering hormone. J. Exp. Biol..

[bib10] Harrison J.F., Greenlee K.J., Verberk W.C.E.P. (2018). Functional hypoxia in insects: definition, assessment, and consequences for physiology, ecology, and evolution. Annu. Rev. Entomol..

[bib11] Horodyski F.M., Ewer J., Riddiford L.M., Truman J.W. (1993). Isolation, characterization and expression of the eclosion hormone gene of Drosophila melanogaster. Eur. J. Biochem..

[bib12] Horodyski F.M., Riddiford L.M., Truman J.W. (1989). Isolation and expression of the eclosion hormone gene from the tobacco hornworm, Manducasexta. Proc. Natl. Acad. Sci. U S A.

[bib13] Ito K., Shinomiya K., Ito M., Armstrong J.D., Boyan G., Hartenstein V., Harzsch S., Heisenberg M., Homberg U., Jenett A. (2014). A systematic nomenclature for the insect brain. Neuron.

[bib16] Kim Y.J., Zitnan D., Cho K.H., Schooley D.A., Mizoguchi A., Adams M.E. (2006). Central peptidergic ensembles associated with organization of an innate behavior. Proc. Natl. Acad. Sci. U S A.

[bib17] Kim Y.J., Zitnan D., Galizia C.G., Cho K.H., Adams M.E. (2006). A command chemical triggers an innate behavior by sequential activation of multiple peptidergic ensembles. Curr. Biol..

[bib18] Kingan T.G., Gray W., Zitnan D., Adams M.E. (1997). Regulation of ecdysis-triggering hormone release by eclosion hormone. J. Exp. Biol..

[bib19] Kivela S.M., Friberg M., Wiklund C., Leimar O., Gotthard K. (2016). Towards a mechanistic understanding of insect life history evolution: oxygen-dependent induction of moulting explains moulting sizes. Biol. J. Linn. Soc..

[bib20] Kruger E., Mena W., Lahr E.C., Johnson E.C., Ewer J. (2015). Genetic analysis of Eclosion hormone action during Drosophila larval ecdysis. Development.

[bib21] Lakes-Harlan R., Pollack G.S., Merritt D.J. (1991). From embryo to adult - anatomy and development of a leg sensory organ in phormia-reginameigen (insecta, diptera) .1.anatomy and physiology of a larval leg sensory organ. J. Comp. Neurol..

[bib23] McGuire S.E., Le P.T., Osborn A.J., Matsumoto K., Davis R.L. (2003). Spatiotemporal rescue of memory dysfunction in Drosophila. Science.

[bib24] McKay D.J., Estella C., Mann R.S. (2009). The origins of the Drosophila leg revealed by the cis-regulatory architecture of the Distalless gene. Development.

[bib25] McNabb S.L., Baker J.D., Agapite J., Steller H., Riddiford L.M., Truman J.W. (1997). Disruption of behavioral sequence by targeted death of peptidergic neurons in *Drosophila*. Neuron.

[bib26] McNabb S.L., Truman J.W. (2008). Light and peptidergiceclosion hormone neurons stimulate a rapid eclosion response that masks circadian emergence in Drosophila. J. Exp. Biol..

[bib27] Miyan J.A. (1989). The thoracic mechanism for eclosion and digging during the extrication behavior of diptera. Physiol. Entomol..

[bib28] Nern A., Pfeiffer B.D., Rubin G.M. (2015). Optimized tools for multicolor stochastic labeling reveal diverse stereotyped cell arrangements in the fly visual system. Proc. Natl. Acad. Sci. U S A.

[bib30] Park Y., Filippov V., Gill S.S., Adams M.E. (2002). Deletion of the ecdysis-triggering hormone gene leads to lethal ecdysis deficiency. Development.

[bib31] Park Y., Zitnan D., Gill S.S., Adams M.E. (1999). Molecular cloning and biological activity of ecdysis-triggering hormones in Drosophila melanogaster. FEBS Lett..

[bib32] Roller L., Zitnanova I., Dai L., Simo L., Park Y., Satake H., Tanaka Y., Adams M.E., Zitnan D. (2010). Ecdysis triggering hormone signaling in arthropods. Peptides.

[bib33] Sweeney S.T., Broadie K., Keane J., Niemann H., O'kane C.J. (1995). Targeted expression of tetanus toxin light chain in Drosophila specifically eliminates synaptic transmission and causes behavioral defects. Neuron.

[bib34] Truman J.W. (2005). Hormonal control of insect ecdysis: endocrine cascades for coordinating behavior with physiology. Vitam. Horm..

[bib35] Truman J.W., Copenhaver P.F. (1989). The larval eclosion hormone neurons in manduca-sexta - identification of the brain-proctodealneurosecretory-system. J. Exp. Biol..

[bib36] Truman J.W., Riddiford L.M. (1970). Neuroendocrine control of ecdysis in silkmoths. Science.

[bib37] Truman J.W., Taghert P.H., Copenhaver P.F., Tublitz N.J., Schwartz L.M. (1981). Eclosion hormone may control all ecdyses in insects. Nature.

[bib38] White B.H., Ewer J. (2014). Neural and hormonal control of postecdysial behaviors in insects. Annu. Rev. Entomol..

[bib39] Whitten J.M. (1957). The post-embryonic development of the tracheal system in Drosophila-melanogaster. Q. J. Microsc. Sci..

[bib40] Zitnan D., Adams M.E., Gilbert L.I. (2012). Neuroendocrine regulation of ecdysis. Insect Endocrinology.

[bib41] Zitnan D., Kingan T.G., Hermesman J.L., Adams M.E. (1996). Identification of ecdysis-triggering hormone from epitracheal endocrine system. Science.

