# Supplementary material for: Non-canonical Eclosion Hormone-Expressing Cells Regulate Drosophila Ecdysis
Source: iScience. 2020 Apr 27;23(5):101108. doi: 10.1016/j.isci.2020.101108 (PMC7225733; doi:10.1016/j.isci.2020.101108)
Supplement: Document S1. Transparent Methods, Figures S1, and Table S1 [file mmc1.pdf]

iScience, Volume *23*

Supplemental Information

Non-canonical Eclosion Hormone-Expressing Cells Regulate *Drosophila* Ecdysis

Robert L. Scott, Fengqiu Diao, Valeria Silva, Sanghoon Park, Haojiang Luan, John Ewer, and Benjamin H. White

Supplemental Figures:

Fig. S1: Trojan exon lines targeting the *Eh* gene and the aEH$_C$ **antibody recognizes novel EH-expressing neurons in adult *Drosophila*. (**Related to Fig.s 1 and 3)

(**A**) Constructs used to target *Eh*-expressing cells in *Drosophila*. *Eh^{ups}-Gal4* (top) is a previously described promoter fusion construct in which Gal4 expression is driven by 2.4 kb of regulatory sequence directly 5' of the *Eh* coding sequence (blue line). *Eh^{TGEM}-Gal4* contains a Trojan Gal4 Expression Module (TGEM; Diao et al., 2015) in the 3rd intron of the *Eh* gene. This construct is flanked by attP sites, which were used to replace the TGEM with two further constructs using ΦC31 integrase. These constructs contain the sequence of the 3rd intron 3' of the insertion site (red arrowhead) and the coding sequence of *Eh* Exon 4 immediately followed by sequences encoding either *T2A-Gal4* or *T2A-p65AD*. Unlike *Eh^{TGEM}-Gal4*, which truncates the *Eh* gene product, these constructs, *Eh^{pan}-Gal4* and *Eh^{pan}-p65AD*, produce full-length EH (fused at the C-terminus with the viral T2A peptide) plus either Gal4 or the Split Gal4 component p65AD.

(**B**) Expression pattern of *Eh^{TGEM}-Gal4* in the pharate adult brain revealed by a UAS-*6xGFP* reporter (green). In addition to the V_m neurons, this line also labels the D_l and D_m groups. Blue, nc82 labeling of neuropil. Scale bars in all images: 100μm.

(**C**) Pharate adult brain expression of the Split Gal4 *Eh^{pan}-p65AD* hemidriver in combination with an *elav-Gal4DBD*. Top, merged image of UAS-*GFP* reporter expression (green), aEH$_C$ immunostaining (red), and neuropil labeling by anti-nc82 (blue). Middle and bottom panels show GFP and aEH$_C$ labeling alone to reveal overlap in the V_m, D_l, and D_m neurons.

(**D**) Anti-EH immunostaining with the aEH$_C$ antibody (red) labels both V_m and D_l neurons in the pharate adult brain of a wildtype, Canton-S animal (top panel). No labeling, however is seen in the brain from an *Eh* null mutant (*Df(3)Eh/Eh^{exc}*) in which growth to the pharate adult stage has been rescued by expression of a UAS-*Eh* transgene in the Inka cells using the *ETH-Gal4* driver.

(**E**) Anti-EH immunostaining (red) persists in D_l but not V_m neurons in 1-4 d old Canton-S flies. Blue, nc82 labeling of neuropil.

Figure S1

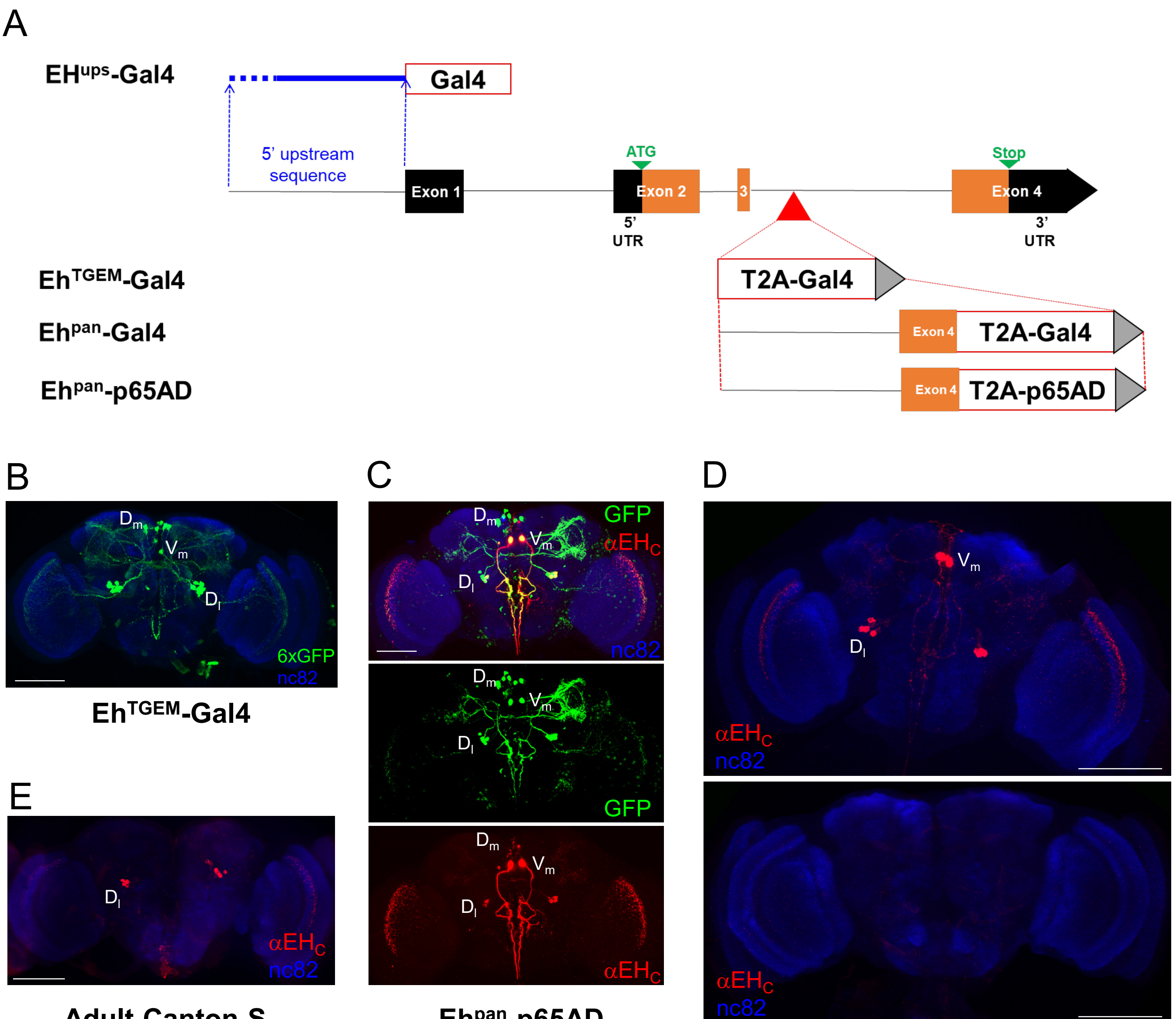

Adult Canton-S

Eh^pan-p65AD ∩ elav-Gal4DBD

ETH-Gal4>UAS-Eh Df(3)Eh/Eh^exc

Supplemental Table S1: Related to Figures 1-4 and Supplemental Figure S1
Listed are the Key Resources (reagents and fly lines) used to generate the data in this study

REAGENT or RESOURCE	SOURCE	IDENTIFIER
**Antibodies**		
Mouse anti-Bruchpilot (1:50 dilution)	Developmental Studies Hybridoma Bank	Cat# nc82, RRID:AB_2314866
Chicken anti-GFP (1:500 dilution)	ThermoFisher	Cat# A10262, RRID:AB_2534023
Rat anti-FLAG-Tag	Novus Biologicals	Cat# NBP1-06712, RRID:AB_1625981
Mouse anti-V5-Tag	Bio-Rad	Cat# MCA1360GA, RRID:AB_567249
Rabbit anti-HA-Tag	Cell Signaling Technology	Cat# 3724, RRID:AB_1549585
Rabbit anti-EH c-terminal (1:5000 dilution)	This paper	
Goat anti-chicken Alexa Fluor 488 (1:500 dilution)	ThermoFisher	Cat# A32931, RRID: AB_2762843
Goat anti-rat Alexa Fluor 488 (1:500 dilution)	ThermoFisher	Cat# A11006, RRID: AB_2534074
Goat anti-rabbit Alexa Fluor 555 (1:500 dilution)	ThermoFisher	Cat# A21428, RRID: AB_2535849
Goat anti-mouse Alexa Fluor 546 (1:500 dilution)	ThermoFisher	Cat# A11003, RRID: AB_2534071
Goat anti-mouse Alexa Fluor 647 (1:500 dilution)	ThermoFisher	Cat# A21236, RRID: AB_2535805
Goat anti-rabbit Alexa Fluor 680 (1:500 dilution)	ThermoFisher	Cat# A21109, RRID: AB_2535758
**Chemicals, Peptides, and Recombinant Proteins**		
Q5 High-Fidelity DNA Polymerase	New England Biolabs	Cat# M0491L
Vectashield	Vector Labs	Cat# H-1000-10
**Critical Commercial Assays**		
SuperScript IV Reverse Transcriptase Kit	ThermoFisher	Cat# 18091050
Zymoclean Gel DNA Recovery Kit	Zymo	Cat# D4001
**Experimental Models: Organisms/Strains**		
D. melanogaster: Eh^{ups}-Gal4	Gift of J. Truman	
D. melanogaster: Eh^{pan}-Gal4	This paper	
D. melanogaster: Eh^{pan}-$P65AD^{16e}$	This paper	
D. melanogaster: Eh^{TGEM}-Gal4	This paper	
D. melanogaster: UAS-CD4::tdGFP	Bloomington Drosophila Stock Center (BDSC)	RRID: BDSC_35836
D. melanogaster: Multi-Color Flip-out	BDSC	RRID: BDSC_64085
D. melanogaster: Df(3)Eh	(Kruger et al., 2015)	
D. melanogaster: Eh^{exc}	(Kruger et al., 2015)	
D. melanogaster: Canton-S	BDSC	
D. melanogaster: ETHRA-Gal4	(Diao et al., 2016)	
D. melanogaster: ETHRB-Gal4	(Diao et al., 2016)	

D. melanogaster: ETH-Gal4	(Diao et al., 2015)	
D. melanogaster: UAS-EH	(Kruger et al., 2015)	
D. melanogaster: 2xUAS-Kir2.1	(Diao et al., 2015)	
D. melanogaster: TNT-E	Gift of G. Davis	
D. melanogaster: w^{1118}	BDSC	
D. melanogaster: elav-Gal4DBD	(Luan et al., 2006b)	
D. melanogaster: tubP-Gal4DBD	(Diao et al., 2015)	
D. melanogaster: UAS-rpr	BDSC	RRID:BDSC_50791
D. melanogaster: P{EH}	(Kruger et al., 2015)	
D. melanogaster: esg-sfGFP	BDSC	RRID:BDSC_78334
**Oligonucleotides**		
EH-1 F (CACACCTTTGCTGCCAAACA)	This paper	
EH-1 R (GTGGTCGATATCAGCAGCCA)	This paper	
EH-2 F (CTTGATTTTGTGCACCT)	This paper	
EH-2 R (CTATATCCTCGCAGTCC)	This paper	
HJ001 (AGTCAGGGTCTCACTAGTGAACTTTAGGAAGTAAATGCC)	This paper	
HJ002 (AGTCAGGGCGCGCCATTTAAATATTAATATCCTTAAAAGTTCC)	This paper	
HJ003 (AGTCAGGCGGCCGCCTCAGGATCAAATGAATGC)	This paper	
HJ004 (AGTCAGACCGGTGTTTCTTGGGGATTTGCCATTATG)	This paper	
**Recombinant DNA**		
Plasmid: pT-GEM(0)	(Diao et al., 2016)	RRID: Addgene_62891
Plasmid: pT-GEM(0)-*Eh*	This paper	
Plasmid: pBS-KS-attb-*Eh*[intron3-exon4)-T2A-Gal4-pA	This paper	
Plasmid: pBS-KS-attb-*Eh*[intron3-exon4)-T2A-P65AD-pA	This paper	
**Software**		
Fiji	(Schindelin et al., 2012)	https://fiji.sc
CMTK Registration plugin for Fiji	(Ostrovsky et al., 2013)	https://www.nitrc.org/projects/cmtk/
JFRC2 reference brain	(Jenett et al., 2012)	http://flybrain.mrc-lmb.cam.ac.uk/si/bridging/www/brains/

Supplemental Table S2: Related to Figures 1-4 and Supplemental Figure S1
Shown are the parental genotypes used to generate the animals shown in each figure panel.

Figure	Females	Males
1A	w ; + ; UAS-CD4::tdGFP	w ; Eh^{ups}-Gal4 ; +
1B	w ; + ; UAS-CD4::tdGFP	w ; + ; Eh^{pan}-Gal4
1C	CantonS	CantonS
1D	w ; + ; UAS-CD4::tdGFP	w ; + ; Eh^{pan}-Gal4
1E-1K	w hs-FLP 2C; + ; UAS-MCFO-1	w ; + ; Eh^{pan}-Gal4
1L	w ; + ; UAS-CD4::tdGFP	yw UAS-eYFP ; Sp/Cy ; ETHRA-T2A-Gal4[m7-1]
1M	w ; + ; UAS-CD4::tdGFP	yw UAS-eYFP ; Sp/Cy ; ETHRB-T2A-Gal4[m67-2]
2A	w ; + ; Eh^{pan}-Gal4	w ; UAS-Kir2.1 ; UAS-Kir2.1
	w ; + ; Eh^{pan}-Gal4	w ; + ; UAS-TNT-E
	w ; + ; Eh^{pan}-Gal4	w ; + ; +
	w ; Eh^{ups}-Gal4 ; +	w ; UAS-Kir2.1 ; UAS-Kir2.1
	w ; Eh^{ups}-Gal4 ; +	w ; + ; UAS-TNT-E
	w ; Eh^{ups}-Gal4 ; +	w ; + ; +
2B	w ; ETH-Gal4 ; Df(3)Eh/TM3 Sb	w ; UAS-EH ; Eh^{exc}/TM3 Ser Act-GFP
2C (N,O,P)	w ; ETH-Gal4 ; Df(3)Eh/TM3 Sb	w ; UAS-EH ; Eh^{exc}/TM3 Ser Act-GFP
2C (E)	Canton-S	Canton-S
3A-3D	w ; + ; UAS-CD4::tdGFP	w ; + ; Eh^{pan}-Gal4
3E	w ; + ; Eh^{pan}-P65AD^{16e}/TM3 Sb	y w ; elav-Gal4DBD; UAS-Kir2.1
	w ; + ; Eh^{pan}-P65AD^{16e}/TM3 Sb	y w UAS-eYFP ; UAS-Kir2.1 ; tubP-Gal4DBD
3F-3K, 3M	w ; + ; UAS-CD4::tdGFP	w ; + ; Eh^{pan}-Gal4
3L	w ; + ; Eh^{pan}-Gal4	w ; esg-sfGFP, UAS-mCherry/Cy ; +
3N	w ; + ; UAS-CD4::tdGFP	w ; Eh^{ups}-Gal4 ; +
3O (left lane)	Canton-S	Canton-S
3O (right lane)	w ; + ; Eh^{exc}/TM3 Ser Act-GFP	w ; + ; Df(3)Eh/TM3 Sb
4A	w ; + ; Eh^{pan}-Gal4	w ; + ; UAS-rpr
4B-4E	w ; + ; +	w ; + ; UAS-rpr
	w ; Eh^{ups}-Gal4 ; +	w ; + ; UAS-rpr
	w ; + ; Eh^{pan}-Gal4	w ; + ; UAS-rpr
	w ; + ; Eh^{exc}/TM3 Ser Act-GFP	w ; + ; Df(3)Eh/TM3 Sb
S1B	w ; + ; UAS-6xGFP	w ; + ; Eh^{TGEM}-Gal4
S1C	w ; + ; Eh^{pan}-P65AD^{16e}/TM3 Sb	y w ; elav-Gal4DBD; UAS-eGFP
S1D (top)	CantonS	CantonS
S1D (bottom)	w ; ETH-Gal4 ; Df(3)Eh/TM3 Sb	w ; UAS-EH ; Eh^{exc}/TM3 Ser Act-GFP
S1E	Canton-S	Canton-S

Transparent Methods

Fly lines
Flies were raised on Nutri-Fly BF food (Genesee Scientific, El Cajon, CA) and maintained at 25°C/50% relative humidity on a constant 12 h light/dark cycle unless otherwise noted. For all transgenic fly lines generated for this study, embryo injections were performed by Rainbow Transgenic Flies, Inc (Camarillo, CA). All other lines are listed in Supplemental Table 1 describing Key Resources.
The Eh^{TGEM}-*Gal4* line was made by inserting a Trojan Gal4 Expression Module (TGEM) (Diao et al., 2015) into Intron 3 of the *Eh* gene using CRISPR/Cas9 at the following cleavage site: GATATTAATATTTAAATCTCAGG (PAM site underlined). To make the TGEM construct, homologous arms of 1 kb flanking the Cas9 cleavage site were amplified by PCR using the following primer pairs: HJ001/HJ002 (agtcagggtctcactagtgaactttaggaagtaaatgcc/agtcagggcgcgccatttaaatattaatatccttaaaagttcc), and HJ003/HJ004 (agtcaggcggccgcctcaggatcaaatgaatgc/agtcagaccggtgtttcttggggatttgccattatg) (Integrated DNA Technologies, Inc., Coralville, Iowa, USA). The PCR products were cloned into the pT-GEM(0) vector. The resulting pT-GEM(0)-*Eh* plasmid was co-injected with a pBS-U6-sgRNA-*Eh* plasmid encoding the guide RNA into embryos of flies expressing germline Cas9 as described previously (Diao et al., 2015). Transformants were identified by their expression of the 3xP3-RFP marker. The Eh^{TGEM}-*Gal4* line was strongly and unexpectedly mutagenic even in heterozygous animals, which resulted in considerable developmental lethality. Reasoning that mutagenicity might result from truncation of the EH protein, we created alternative constructs (Eh^{pan}-*Gal4* and Eh^{pan}-*P65AD*) for insertion into the same locus using □C31-mediated integration into the *attP* landing sites flanking the TGEM insert of the Eh^{TGEM}-*Gal4* line. For both of these cases, pBS vectors contained *attB* sites flanking DNA from the intron sequence downstream of the original TGEM insertion to the 3' end of exon 4, followed by a GGGGS linker and either *T2A-Gal4*-pA or *T2A-P65AD*-pA (Synthesized by Epoch Life Science, Inc., Missouri City, TX). Transformants were identified by the loss of 3xP3-*RFP* marker expression.

Antibody production
Peptide corresponding to the carboxy terminus of EH (CEDIASIAPFLNALE) was synthesized by Bethyl Laboratories (Montgomery, TX) and conjugated to KLH through the N-terminal cysteine. After screening serum from two rabbits immunized with the conjugated peptide, the hyperimmune serum from both rabbits was pooled and affinity purified using peptide coupled to agarose.

RT-PCR
Total RNA was prepared from dissected trachea (including the posterior spiracles and dorsal trunks with attached tracheal branches) of first and second instar larvae. After dissection in PBS, tracheal RNA was isolated using Trizol reagent (ThermoFisher, Waltham, MA) and then DNAse I treated. Approximately ~0.5μg was used for first strand cDNA synthesis primed by oligo dT (SuperScript IV Reverse Transcriptase Kit, ThermoFisher). PCR was performed using Q5 DNA polymerase (NEB, Ipswich, MA) and the following primer pairs at the indicated annealing temperatures: EH-1 F/R (cacaccttgctgccaaaca)/ (gtggtcgatatcagcagcca) at 66˚C, EH-2 F/R (cttgattttgtgcacct)/ (ctatatcctcgcagtcc) at 58˚C, and rp49 F/R (cggatcgatatgctaagctgt)/ (gcgcttgttcgatccgta) at 65˚C using a Bio-Rad C1000 Touch thermocycler (Bio-Rad, Hercules, CA). Products were separated on 3% agarose gel and stained with GelRed (Biotium, Fremont, CA). Bands were isolated and purified (Zymo Gel DNA Recovery Kit) before being sequenced (Psomagen, Rockville, MD).

Immunohistochemistry
Nervous system whole mounts were excised from L1-L3 larvae or pharate adults and prepared for immunolabeling as described previously, using 5% normal goat serum in the blocking solution (Luan et al., 2006a). Rabbit αEH_C was used at 1:5000 dilution. Neuropil was visualized using mouse anti-Brp (1:50; Developmental Studies Hybridoma Bank, mAb nc82) and anti-GFP (1:500, ThermoFisher #A10262). Secondary antibodies were used at 1:500 (anti-chicken Alexa Fluor 488, anti-rabbit Alexa Fluor 555, and anti-mouse Alexa Fluor 647; ThermoFisher, Waltham, MA). Multi-Color Flip Out labeling followed the procedure reported by (Nern et al., 2015). MCFO-1 with hs-FLP-PEST females were crossed with Eh^{pan}-*Gal4* males. The progeny were raised at 18˚C until the pharate adult stage before being heat-shocked in a 37˚C water bath for 15–30 min to label single neurons. After heat shock, the animals were incubated at 25˚C overnight before dissecting out the brains. For the immunostaining, brains were

incubated with primary antibodies: anti-FLAG (rat, 1:200, Novus Biological Inc), anti-V5 (mouse, 1:300, Bio-Rad), and anti-HA (rabbit, 1:300, Cell Signaling Technology, Inc) at room temperature overnight. After washing with PBS, brains were incubated in secondary antibodies (anti-mouse Alex Fluor 546, anti-rat Alex Fluor 488, and anti-rabbit Alex Fluor 680) (ThermoFisher, Waltham, MA) at 4˚C overnight.

Microscopy
CNS imaging was done using a Nikon C2 confocal microscope with a 20X air objective. Unless otherwise noted, the images presented are maximum intensity projection images of a Z-stack collected through the entire preparation. Brain alignment for anatomical localization of projections was performed using the CMTK Registration Plugin for Fiji (Ostrovsky et al., 2013) against the Janelia Reference Brain - JFRC2 (Jenett et al., 2012). Imaging of live larva was done on a Nikon Eclipse Ti wide-field microscope using 10x or 20x air objectives. Larva were chosen immediately after shedding their first instar cuticle and were anesthetized using diethyl ether (Kakanj et al., 2020) before being mounted in Vectashield (Vector Labs, Burlingame, CA) with a 0.17mm spacer. The red and green channels in Fig. 3L were exchanged in order to maintain color consistency for EH labeling in the figure.
Video recording was done using a Sony NEX-VG20 HandyCam (Sony Corp., Tokyo, Japan) mounted on an Olympus SZX-16 stereomicroscope (Olympus, Center Valley, PA) with diffused white light. Animals to be video recorded were chosen from the culture vial prior to ptilinum inflation and transferred to a slide on a 25˚C constant temperature block. Flies were recorded until eclosion, or until 2 hours had passed since the start of ptilinum extension.

Developmental lethality and adult ecdysis assays
Developmental lethality was assayed as follows in crosses of *w;*UAS-*Kir2.1;*UAS-*Kir2.1* males to either *w;+;+, w;*Eh^{ups}*-Gal4;+*, or *w;+;*Eh^{pan}*-Gal4* virgin females: mated females were allowed to lay for up to six hours in food vials before being removed. Eggs were counted and then vials were incubated at 25˚C for at least 14 days after which dead pupae and emerged adults were counted. Adults were scored positive for wing expansion if their wings exhibited either a cupped or flat appearance.
The effects of adult-specific neuronal suppression with either 2X UAS-*Kir2.1* or 1X UAS-*TNT-E* were carried out with the ubiquitously expressed, temperature-sensitive *Gal4* inhibitor, *tub-Gal80ts*. Animals were grown at 18°C, collected as wandering 3rd instar larvae and then incubated for a further 6 d at 18°C before being shifted to 31°C until eclosion. Any flies that failed to completely emerge from their pupal cases or fully expand their wings after this time were scored as “Eclosion failure” or “Non-expanded,” respectively.
For split-Gal4 crosses, Eh^{pan}*-P65AD/TM3 Sb* virgin females and either *w;elav-DBD;UAS-Kir2.1* or *UAS-Kir2.1;tubP-DBD* males were allowed to lay for 3-4 days before adults were removed. Emerged adults were counted and scored for the presence of the *Sb* marked balancer.
For the *ETH-Gal4*>UAS-*Eh* rescue experiments, *w;ETH-Gal4;Df(3)Eh/TM3 Sb* virgin females were crossed to *w;*UAS-*Eh;*Eh^{exc}*/TM3 Ser Act-GFP* males and allowed to lay for 3-6 days. At least five days after the first flies emerged, adults were scored for eclosion failure.

Larval ecdysis behavior
Larvae were collected and their ecdysis behaviors recorded as described previously (Clark et al., 2004, Park et al., 2002). Briefly, larvae were reared at 25˚C on Petri dishes with fly medium. First instar larvae approaching ecdysis to the second instar were identified by their double mouth hooks, placed on Petri dishes with agar, and video-recorded starting when they first pigmented their double vertical plates (DVP stage; ca. 30 min away from ecdysis). Animals were recorded until they completed ecdysis or for up to 2h after the time of ecdysis of control animals. Behavioral analysis scored for the presence or absence of three different phases: “locomotion,” consisting of normal locomotor activity; “pre-ecdysis,” consisting of anterior-posterior contractions (AP) and squeezing waves (SW); and “ecdysis” which started with biting behavior and ended with the final backward thrust, regardless of whether the old cuticle was eventually shed. “Atypical pre-ecdysis” was characterized by partial, weak or missing AP contractions or SW. Tracheal measurements in each larva included: (1) the time from the beginning of tracheal collapse to the beginning of air filling, (2) the percentage of animals that completed air filling, and (3) the time taken to completely fill the trachea, for those animals that successfully completed that process.

Supplemental References

CLARK, A. C., DEL CAMPO, M. L. & EWER, J. 2004. Neuroendocrine control of larval ecdysis behavior in Drosophila: complex regulation by partially redundant neuropeptides. *J Neurosci,* 24**,** 4283-92.

DIAO, F., IRONFIELD, H., LUAN, H., DIAO, F., SHROPSHIRE, W. C., EWER, J., MARR, E., POTTER, C. J., LANDGRAF, M. & WHITE, B. H. 2015. Plug-and-Play Genetic Access to Drosophila Cell Types using Exchangeable Exon Cassettes. *Cell Reports,* 10**,** 1410-1421.

DIAO, F. C., MENA, W., SHI, J., PARK, D., DIAO, F. Q., TAGHERT, P., EWER, J. & WHITE, B. H. 2016. The Splice Isoforms of the Drosophila Ecdysis Triggering Hormone Receptor Have Developmentally Distinct Roles. *Genetics,* 202**,** 175-189.

JENETT, A., RUBIN, G. M., NGO, T. T. B., SHEPHERD, D., MURPHY, C., DIONNE, H., PFEIFFER, B. D., CAVALLARO, A., HALL, D., JETER, J., IYER, N., FETTER, D., HAUSENFLUCK, J. H., PENG, H. C., TRAUTMAN, E. T., SVIRSKAS, R. R., MYERS, E. W., IWINSKI, Z. R., ASO, Y., DEPASQUALE, G. M., ENOS, A., HULAMM, P., LAM, S. C. B., LI, H. H., LAVERTY, T. R., LONG, F. H., QU, L., MURPHY, S. D., ROKICKI, K., SAFFORD, T., SHAW, K., SIMPSON, J. H., SOWELL, A., TAE, S., YU, Y. & ZUGATES, C. T. 2012. A GAL4-Driver Line Resource for Drosophila Neurobiology. *Cell Reports,* 2**,** 991-1001.

KAKANJ, P., EMING, S. A., PARTRIDGE, L. & LEPTIN, M. 2020. Long-term in vivo imaging of Drosophila larvae. *Nature Protocols,* 15, 1158–1187.

KRUGER, E., MENA, W., LAHR, E. C., JOHNSON, E. C. & EWER, J. 2015. Genetic analysis of Eclosion hormone action during Drosophila larval ecdysis. *Development,* 142**,** 4279-4287.

LUAN, H., LEMON, W. C., PEABODY, N. C., POHL, J. B., ZELENSKY, P. K., WANG, D., NITABACH, M. N., HOLMES, T. C. & WHITE, B. H. 2006a. Functional dissection of a neuronal network required for cuticle tanning and wing expansion in Drosophila. *J Neurosci,* 26**,** 573-84.

LUAN, H., PEABODY, N. C., VINSON, C. R. & WHITE, B. H. 2006b. Refined spatial manipulation of neuronal function by combinatorial restriction of transgene expression. *Neuron,* 52**,** 425-36.

NERN, A., PFEIFFER, B. D. & RUBIN, G. M. 2015. Optimized tools for multicolor stochastic labeling reveal diverse stereotyped cell arrangements in the fly visual system. *Proceedings of the National Academy of Sciences of the United States of America,* 112**,** E2967-E2976.

OSTROVSKY, A., CACHERO, S. & JEFFERIS, G. 2013. Clonal analysis of olfaction in Drosophila: image registration. *Cold Spring Harb Protoc,* 2013**,** 347-9.

PARK, Y., FILIPPOV, V., GILL, S. S. & ADAMS, M. E. 2002. Deletion of the ecdysis-triggering hormone gene leads to lethal ecdysis deficiency. *Development,* 129**,** 493-503.

SCHINDELIN, J., ARGANDA-CARRERAS, I., FRISE, E., KAYNIG, V., LONGAIR, M., PIETZSCH, T., PREIBISCH, S., RUEDEN, C., SAALFELD, S., SCHMID, B., TINEVEZ, J. Y., WHITE, D. J., HARTENSTEIN, V., ELICEIRI, K., TOMANCAK, P. & CARDONA, A. 2012. Fiji: an open-source platform for biological-image analysis. *Nature Methods,* 9**,** 676-682.
